# Trends and Disparities in Deaths from Kidney Disease Among Older Adults in the United States

**DOI:** 10.3390/jcm14144950

**Published:** 2025-07-12

**Authors:** Benjamin Grobman, Connor P. Bondarchuk, Arian Mansur, Christine Y. Lu

**Affiliations:** 1 Harvard Medical School, Boston, MA 02115, USA; 2 Department of Population Medicine, Harvard Pilgrim Health Care Institute, Boston, MA 02215, USA; 3 School of Pharmacy, Faculty of Medicine and Health, The University of Sydney, Sydney, NSW 2050, Australia; 4 Kolling Institute, Faculty of Medicine and Health, The University of Sydney and the Northern Sydney Local Health District, Sydney, NSW 2065, Australia

**Keywords:** geriatric, disparities, kidney, epidemiology

## Abstract

**Background:** Kidney disease is a significant cause of morbidity and mortality in the United States. However, less is known about its burden specifically among older adults. **Methods:** We analyzed deaths among U.S. adults aged 65 and older between 2018 and 2023 where kidney disease was listed as the primary cause of death, using data from the Centers for Disease Control and Prevention Wide-ranging Online Data for Epidemiologic Research (CDC WONDER). We examined overall and subgroup-specific death counts stratified by sex, race, ethnicity, and geographic location. Trends from 1999 to 2023 were also assessed. **Results:** Between 2018 and 2023, there were 263,436 deaths among adults aged 65+ with kidney disease as the primary cause, accounting for 81.5% of all kidney disease deaths during this period. Mortality rates were significantly higher among males compared to females (age-adjusted mortality rate (AAMR) ratio = 1.42, 95% CI: 1.42–1.43) and among Black Americans compared to White Americans (AAMR ratio = 1.94, 95% CI: 1.93–1.95). From 1999 to 2009, kidney disease mortality rates increased (annual percent change (APC) = 1.40, 95% CI: 0.90, 2.09), declined between 2009 and 2012 (APC = −5.35, 95% CI: −6.59, −2.33), and remained stable from 2012 to 2023. **Conclusions:** This majority of kidney disease deaths in the U.S. occur among older adults. While mortality briefly declined between 2009 and 2012, progress has since stalled. Persistent disparities by race and geography highlight the need for targeted research and interventions to reduce kidney disease mortality among older adults.

## 1. Introduction

Kidney disease is a significant cause of morbidity and mortality in the United States. In 2018, the incidence of end-stage renal disease in the United States was 2242 cases per million population. During that same year, 554,038 patients were receiving dialysis and 229,887 were living with a transplanted kidney [1]. Additionally, in 2016, there were 82,549 deaths from chronic kidney disease among adults in the United States [2]. Older adults are particularly vulnerable to kidney disease and its complications. As the global population continues to age, kidney disease is expected to become the second leading cause of death globally by the end of the 21st century [3]. The burden of kidney disease has increased rapidly in recent years, driven largely by increases in diabetes, the primary risk factor for chronic kidney disease [4]. In the United States, multiple ongoing interventions and advancements in treatment aim to reduce this burden. These include new recommendations for use of sodium/glucose cotransporter 2 inhibitors and glucagon-like peptide 1 receptor agonists in management of chronic kidney disease, as well as health services initiatives to increase access to kidney transplantation [5]. Additionally, ongoing research is exploring new technologies such as porcine xenotransplantation [6,7].

Despite these advances, there remain significant disparities in kidney disease incidence, treatment, and outcomes. Black adults have more than three times the risk of developing end-stage renal disease [8], and people from minority backgrounds with end-stage renal disease are less likely to receive a kidney transplant than White people [9]. Mortality rates from kidney disease among Black Americans are more than double those among White Americans [10]. Hispanic Americans have nearly a 50% higher incidence of end-stage renal disease compared to non-Hispanic White adults [11]. Native Americans have historically experienced end-stage renal disease rates 3.5 times higher than those of White Americans [12].

Importantly, kidney disease not only serves as the primary cause of death but also significantly contributes to other leading causes of mortality. For example, many individuals with kidney disease ultimately die from cancer or heart disease [13]. One study found that as kidney function worsens, the proportions of deaths due to heart failure and infectious diseases increases [13]. Chronic kidney disease leads to systemic inflammatory changes that contribute to vascular remodeling and damage, which in turn elevates the risk of cardiovascular complications [14]. Additionally, impaired renal function increases the likelihood of developing hypertension, the leading global risk factor for cardiovascular disease, creating a bidirectional relationship between chronic kidney disease and cardiovascular disease [15,16]. As a result, cardiovascular disease is the leading cause of death among people with end-stage renal disease, rather than end-stage renal disease itself [14]. These interconnections underscore the need to examine kidney disease both as the primary and a contributing cause of death in order to fully assess its public health impact.

Despite the increasing burden and well-documented disparities in kidney disease, less is known about the epidemiology of kidney disease deaths among older adults across demographic subgroups such as race, ethnicity, and geographic region. This study addresses these gaps. We examined mortality from kidney disease among U.S. older adults aged 65 and over from 2018 to 2023, considering both primary and contributing causes of death. We also analyzed trends in kidney disease mortality from 1999 to 2023. By identifying patterns and disparities, our goal is to inform the development of targeted interventions to reduce kidney disease mortality in older and at-risk populations.

## 2. Materials and Methods

### 2.1. Data Source

This study used data from the Multiple Cause of Death database within the Centers for Disease Control and Prevention’s Wide-ranging Online Data for Epidemiologic Research (CDC WONDER) [17]. This database compiles information from death certificates for all decedents in the 50 U.S. states and the District of Columbia. For cross-sectional analyses, we used the most recent version of the database covering the years 2018–2023. The database contains one primary cause of death from each death certificate and up to 20 additional causes, along with demographic data [17]. To assess long-term trends, we also used an earlier version of the database containing data from 1999–2020. Of note, the 2018–2023 CDC WONDER dataset classifies race into 6 categories (American Indian or Aslaka Native, Asian, Black or African American, Native Hawaiian or Other pacific Islander, White, and more than one race) [18], whereas earlier versions classified race into 4 categories (American Indian or Alaska Native, Asian or Pacific Islander, Black or African American, and White) [17]. Due to these changes in racial categorization, direct comparisons over time were limited. However, we verified that estimates for overlapping years for Black, White and Asian decedents were largely consistent, allowing for meaningful trend analyses within those groups.

### 2.2. Study Population

We identified deaths where kidney disease was listed as either the primary or a contributing cause of death using International Classification of Diseases (ICD)-10 codes N00-N07, N17-N19, and N25-N27, which are consistently used across CDC WONDER datasets. We obtained mortality estimates overall and stratified by sex (male and female), race (White, Black or African American, Asian, American Indian or Alaska Native, Native Hawaiian or Other Pacific Islander, and more than one race), ethnicity (Hispanic vs. non-Hispanic), urbanization level (large central metro, large fringe metro, medium metro, micropolitan, noncore, and small metro), and region (Northeast, Midwest, South, and West). We also examined the 6 leading primary causes of death in people with kidney diseases listed as a contributing cause of death, using CDC’s selected list of 113 primary causes of death.

### 2.3. Statistical Analysis

To assess trends in mortality from 1999 to 2023, we applied Joinpoint Regression analysis using software developed by the National Cancer Institute [19]. This method identifies points (“joinpoints”) at which statistically significant changes in trend occur and estimates the annual percent change (APC) for each time segment [19]. Due to limitations in historical data, trend analyses were restricted to subgroups by sex (male or female), race (Black, White, or Asian), and region (Northeast, Midwest, South, or West).

We calculated age-adjusted mortality rates (AAMRs) using the CDC WONDER interface and calculated AAMR ratios to compare mortality across demographic subgroups. 95% confidence intervals (CI) for AAMR ratios were determined by taking the standard error for group 1 (e.g., Black adults) divided by the mortality rate for group 1 and squaring this term. This was then added to the standard error divided by the AAMR of group 2 squared (e.g., White). We then took the square root of this combined term to produce the standard error for the AAMR ratio (e.g., sqrt((Black standard error/Black AAMR)^2 + (White standard error/White AAMR)^2)) [20]. We then used the qnorm function in R studio to determine the 95% CIs for the AAMR based on this calculated standard error [21]. AAMRs were obtained directly from the CDC WONDER interface while AAMR ratios were calculated using R studio version 4.2.2 (Vienna, Austria) [21]. Figures were generated using the ggplot2 package in R for data visualization [22]. Trends analyses were conducted using Joinpoint version 5.3.0.0 (National Cancer Institute, Bethesda, MD) [23].

## 3. Results

### 3.1. Kidney Disease as the Primary Cause of Death

**Figure 1:**
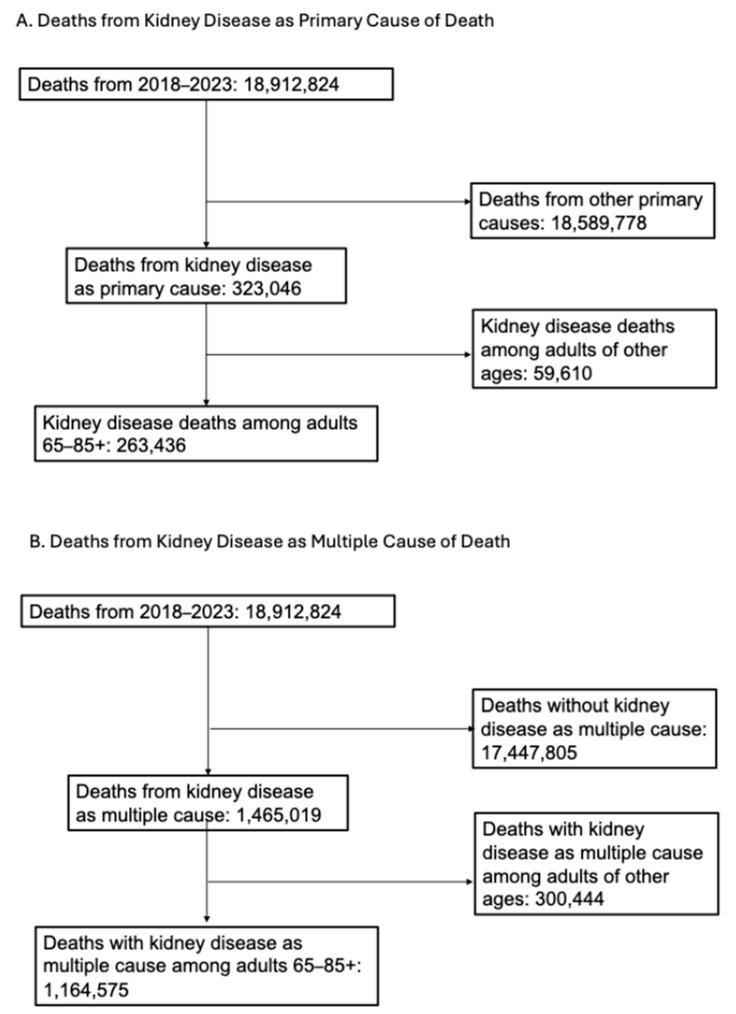
Study inclusion flowchart.

From 2018 to 2023, kidney disease was the primary cause of death in 263,436 adults aged 65 and older (Figure 1), corresponding to an AAMR of 83.61 per 100,000 people (Table 1).

Deaths among adults aged 65 and older accounted for 81.5% of all kidney disease related deaths in the US from 2018–2023. From 1999 to 2009, the overall rate of kidney disease deaths as the primary cause of death increased (APC = 1.40, 95% CI: 0.90, 2.09, *p* < 0.01). A significant decline followed from 2009 to 2012 (APC = −5.35, 95% CI: −6.59, −2.33, *p* < 0.01). From 2012 to 2023, the rate continued to trend downward, though the change was not statistically significant (APC = −0.47, 95% CI: −0.90, 0.40, *p* = 0.20) (Table 2).

**Figure 2:**
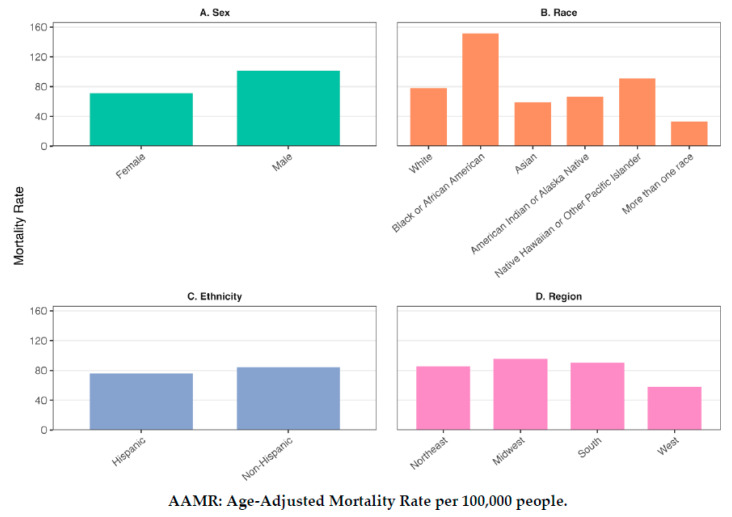
Kidney disease mortality among older adults by sociodemographic subgroup for kidney disease as the primary cause of death. (**A**) Deaths by sex, (**B**) deaths by race, (**C**) deaths by ethnicity, (**D**) deaths by region.

When examining differences by sex, there were 132,944 deaths from kidney disease as the primary case among older adult males (AAMR = 101.497) and 130,492 among older adult females from 2018 to 2023. Death rates were significantly higher among males, with an AAMR ratio of 1.42 (95% CI: 1.42–1.43) (Figure 2). Among males, kidney disease death rates increased from 1999–2009 (APC = 0.97, 95% CI: 0.25, 2.10, *p* = 0.03), decreased significantly from 2009–2012 (APC = −5.23, 95% CI: −6.72, −1.35, *p* = 0.04), and then declined non-significantly from 2012–2023 (APC = −0.68, 95% CI: −1.40, 1.67, *p* = 0.29). Among females, death rates also increased significantly from 1999–2009 (APC = 1.56, 95% CI: 1.09, 2.18, *p* < 0.01), followed by a significant decline from 2009–2012 (APC = −5.72, 95% CI: −6.87, −2.80, *p* < 0.01), and a non-significant decrease from 2012–2023 (APC = −0.43, 95% CI: −0.84, 0.24, *p* = 0.18).

Regarding race, there were 207,548 deaths from kidney disease as the primary cause among White Americans (AAMR = 78.20), 44,155 deaths among Black Americans (AAMR = 151.41), 8794 deaths among Asian Americans (AAMR = 58.61), 1522 deaths among American Indians or Alaska Natives (AAMR = 66.77), 402 deaths among Native Hawaiians or Other Pacific Islanders (AAMR = 402), and 985 deaths among people with more than one race (AAMR = 33.27). Death rates were significantly higher among Black Americans than White Americans (AAMR ratio = 1.94, 95% CI: 1.93–1.95). Higher rates were also observed among Native Hawaiian or Other Pacific Islanders (AAMR ratio = 1.17, 95% CI: 1.07–1.27). In contrast, lower rates were seen among Asian Americans (AAMR ratio = 0.75, 95% CI: 0.73–0.77), American Indians or Alaska Natives (AAMR ratio = 0.85, 95% CI: 0.80–0.91), and people listing more than one race (AAMR ratio = 0.43, 95% CI: 0.36–0.49).

When examining trends over time, kidney disease death rates among White Americans increased from 1999–2009 (APC = 1.54, 95% CI: 0.88, 2.51, *p* = 0.20), followed by a significant decline from 2009–2012 (APC = 5.17, 95% CI: −6.62, −1.55, *p* = 0.03), and a non-significant decrease from 2012–2023 (APC = −0.54, 95% CI: −1.15, 1.37, *p* = 0.32). Similar trends were seen among Black Americans, with rates increasing from 1999–2009 (APC = 0.73, 95% CI: 0.30, 1.36, *p* < 0.01), declining significantly from 2009–2012 (APC = −6.65, 95% CI: −7.80, −3.77, *p* < 0.01), and then showing a non-significant downward trend from 2012–2023 (APC = −0.16, 95% CI: −0.60, 0.50). Among Asian Americans, death rates declined slightly but not significantly from 1999–2023 (APC = −0.33, 95% CI: −0.74, 0.09, *p* = 0.12).

Regarding ethnicity, death rates from kidney disease as the primary cause of death were higher among non-Hispanic older adults (AAMR = 84.12) than among Hispanic adults (AAMR = 75.99) (AAMR ratio = 1.11, 95% CI: 1.01–1.12).

Regarding region, deaths rates from kidney disease as the primary cause of death were higher in the Midwest (AAMR = 95.60) (AAMR ratio = 1.12, 95% CI: 1.11–1.13), the South (AAMR = 90.77) (AAMR ratio = 1.06, 95% CI: 1.05–1.07), compared to the Northeast (AAMR = 85.42), while rates in the West were lower (AAMR = 57.95) (AAMR ratio = 0.68, 95% CI: 0.67–0.69). Trends over time varied by region. In the Northeast, kidney disease death rates increased significantly from 1999–2002 (APC = 2.78, 95% CI: 1.61, 5.15, *p* < 0.01), then declined non-significantly from 2002–2009 (APC = −0.42, 95% CI: −1.26, 0.13, *p* = 0.11). A significant decrease followed from 2009–2012 (APC = −5.59, 95% CI: −6.32, −3.74, *p* < 0.01), and the decline continued non-significantly from 2012–2023 (APC = −0.29, 95% CI: −0.53, 0.06). In the Midwest, rates rose significantly from 1999–2002 (APC = 4.63, 95% CI: 0.88, 10.36, *p* = 0.01), followed by a significant and steady decline from 2002–2023 (APC = −0.82, 95% CI: −1.15, −0.60, *p* < 0.01). In the South, deaths increased significantly from 1999–2009 (APC = 2.48, 95% CI: 1.83, 3.31, *p* < 0.01), then decreased sharply from 2009–2012 (APC = −5.55, 95% CI −6.83, −1.95), followed by a non-significant decrease from 2012–2023 (APC = −1.08, 95% CI: −1.61, 0.25, *p* = 0.07). In the West, deaths increased from 1999–2009 (APC = 1.69, 95% CI: 1.03, 2.70, *p* < 0.01), declined significantly from 2009–2012 (APC = −6.92, 95% CI: −8.61, −2.78, *p* < 0.01), and then increased again from 2012–2023 (APC = 1.41, 95% CI: 0.81, 2.32, *p* < 0.01).

### 3.2. Kidney Disease as a Contributing Cause of Death

**Figure 3:**
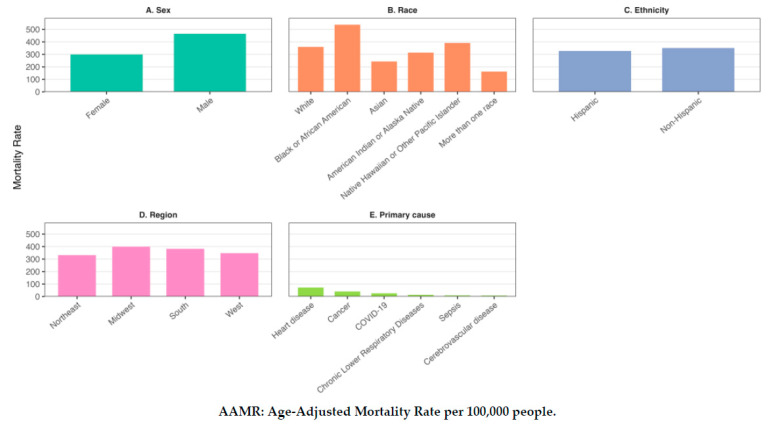
Kidney disease mortality among older adults by sociodemographic subgroup for kidney disease as a multiple cause of death. (**A**) Deaths by sex, (**B**) deaths by race, (**C**) deaths by ethnicity, (**D**) deaths by region (**E**) primary causes of death.

From 2018 to 2023, there were 1,164,575 deaths among older adults aged 65 and older in which kidney disease was listed as a contributing cause of death, corresponding to an AAMR of 369.040 per 100,000 people (Table 3, Figure 3).

There were 551,981 deaths among older adult females (AAMR = 300.52) and 612,594 among males (AAMR = 465.82) in which kidney disease was listed as a contributing cause of death, resulting in an AAMR ratio of 1.55 (95% CI: 1.55–1.55). This indicates substantially higher mortality among males. Racial disparities were also evident. Death rates were significantly higher among Black Americans (AAMR = 537.26) compared to White Americans (AAMR = 359.75) (AAMR ratio = 1.49, 95% CI: 1.49–1.50), and among Native Hawaiian or other Pacific Islanders (AAMR = 1.09, 95% CI: 1.04–1.14) (AAMR ratio = 1.09, 95% CI: 1.04–1.14). In contrast, death rates were lower among Asian Americans (AAMR = 243.27) (AAMR ratio = 0.68, 95% CI: 0.67–0.69), American Indians or Alaska Natives (AAMR = 313.88) (AAMR ratio = 0.87, 95% CI: 0.85–0.90) and among people with more than one race (AAMR = 162.12) (AAMR ratio = 0.45, 95% CI: 0.42–0.48). Similar to patterns seen for kidney disease as a primary cause of death, death rates of kidney disease as a contributing cause of death were higher among non-Hispanic adults (AAMR = 351.56) than Hispanic adults (AAMR = 327.42) (AAMR ratio = 1.07, 95% CI: 1.07–1.08). Regional variations was also observed. Compared to the Northeast (AAMR = 332.11), death rates from kidney disease as a contributing cause of death were higher in the Midwest (AAMR = 399.28) (AAMR ratio = 1.22, 95% CI 1.21–1.23), the South (AAMR = 381.81) (AAMR ratio = 1.17, 95% CI: 1.16–1.17), and the West (AAMR = 347.61) (AAMR ratio = 1.06, 95% CI: 1.05–1.07).

When we examined the leading primary causes of death among people for whom kidney disease was listed as a contributing cause of death, heart disease was the most common underlying cause (AAMR = 72.71), followed by cancer (AAMR ratio = 0.56, 95% CI: 0.56–0.57), COVID-19 (AAMR ratio = 0.37, 95% CI: 0.36–0.38), chronic lower respiratory diseases (AAMR ratio = 0.19, 95% CI: 0.18–0.20), sepsis (AAMR ratio = 0.13, 95% CI: 0.12–0.14), and cerebrovascular diseases (AAMR ratio = 0.12, 95% CI: 0.11–0.13).

## 4. Discussion

This study examined the epidemiology of kidney disease-related mortality (defined as cases where kidney disease was listed as either as a primary or contributing cause of death) among adults in the United States from 2018 to 2023. We found that approximately 81.5% of kidney disease related deaths during this period occurred among people aged 65 and older. We also found that deaths within this age group remained relatively stable over the past decade.

Our findings underscore several key disparities across demographic and geographic groups. For one, males had a 42% higher age-adjusted mortality rate than females for kidney disease as a primary cause of death, and 55% higher when kidney disease was a contributing cause of death. This pattern aligns with prior studies conducted in the United States [24] and globally [25], which have consistently shown lower kidney disease-related mortality among women across age groups and nationalities. The observed survival advantage in females has been attributed to both biological and social factors. Biologically, estrogen is thought to confer reno-protective and vaso-protective effects [26]. Socially, men may be at a disadvantage due to lower rates of care-seeking behavior [27] and a greater burden of comorbidities [27].

In the current analysis, we found that death rates from kidney disease—both as a primary and as a contributing cause of death—were much higher among older Black Americans than among any other racial group. This finding is consistent with a large body of prior research documenting the disproportionate burden of kidney disease among Black Americans in the United States. Although Black Americans represent only 12% of the US population, they account for approximately 27% of kidney disease related deaths [10]. Several factors contribute to this disparity. On average, Black adults in the US have lower socioeconomic status than their white counterparts [28], which is a key determinant of both access to healthcare and chronic disease management. In addition, Black adults also have higher rates of diabetes and hypertension, the two most important risk factors for kidney disease, than other racial and ethnic groups [29,30]. Genetic factors may also play a role; variants in the APOL1 gene, more common among people of African ancestry, have been associated with higher rates of various forms of kidney disease [31]. While there is less research on these disparities specifically in older adults, prior work has similarly shown that older Black adults are at higher risk for developing end-stage renal disease than their White counterparts, findings which are reinforced by the current study [32]. In recent decades, advances in kidney disease treatment, including the introduction of novel reno-protective agents such as SGLT2 and GLP-1 inhibitors, have the potential to reduce overall disease burden and address racial disparities in kidney disease [33]. However, preliminary research suggests that Black patients are less likely than White patients to receive these therapies [34], raising concerns that inequities in treatment access may perpetuate or even widen existing disparities in kidney disease outcomes in the years ahead. Addressing these disparities fully will likely require policy reforms targeting broader socioeconomic inequality in the United States. Nonetheless, despite the limitations of clinical interventions alone, efforts to reduce inequities in healthcare utilization and prescribing practices, along with improved screening and treatment options for APOL1-mediated kidney disease may contribute meaningfully to narrowing racial disparities in kidney disease among older adults.

In terms of ethnicity, we found that kidney disease deaths both as a primary and a contributing cause were lower among Hispanic Americans compared to non-Hispanic Americans. This finding reflects a well-known phenomenon in which Hispanic Americans have better health outcomes despite socioeconomic disadvantages and higher prevalence of risk factors such as obesity and diabetes [35]. Potential explanations include higher levels of social support [36] and healthier profiles among immigrants compared to US-born individuals [37]. However, further research is required to better understand this phenomenon, particularly among older adults.

Geographically, we observed higher mortality rates in the South and Midwest compared to the Northeast for kidney disease as a primary cause of death. The West showed a more complex pattern, with higher rates for kidney disease as a primary cause of death but lower rates for kidney disease as a contributing cause of death. The literature on regional variation in kidney disease remains limited, although previous studies have documented elevated all-cause mortality in the South compared to the rest of the United States [38]. While the reasons for these findings are incompletely elucidated, one major potential contributor is lower average socioeconomic status in the Southern United States [38]. Additionally, poor dietary quality—characterized by high consumption of fried foods and sugar-sweetened beverages, which are common in Southern cuisine—may also play a role in these disparities [38,39]. Such dietary patterns are strongly associated with hypertension, kidney disease, and overall mortality [40]. It is less evident why death rates might be higher in the Midwestern United States. However, given these marked regional disparities, further research should investigate the socioeconomic, clinical, and environmental factors contributing to kidney disease outcomes across different regions. These findings may inform targeted policy interventions to help reduce the burden of kidney disease among older adults.

When examining trends over time, we found that kidney disease mortality increased from 1999 to 2009 across most sociodemographic groups, followed by a significant decline from 2009 to 2012. From 2012 to 2023, death rates trended downward, although these changes were not statistically significant. This is in line with national data showing that the age-adjusted prevalence and crude prevalence of CKD stages 3–4 increased from 1988–1994 to 2003–2004 and then plateaued [41]. The increase in kidney disease mortality from 1999 to 2009 likely reflects broader trends in diabetes and obesity, which surged during this period [42,43]. The causes of these linked epidemics are multifactorial, but these trends have been attributed to an increase in urbanization accompanied by low levels of physical activity and sedentary lifestyles [44]. Sedentary behavior in particular increased in the United Sates during the last half of the 20th century [45]. Although diabetes prevalence rose sharply between 1990 and 2008 in the US, it stabilized thereafter [46]. Initially, improvements in the management of diabetes, hypertension and hyperlipidemia may have contributed to a decline in chronic kidney disease related mortality [47]. However, these improvements began to reverse after 2010 [47], possibly explaining the stagnation in kidney disease mortality reductions in recent years. Further research is required to better understand the trends linking diabetes and CKD. Given that diabetes is the leading cause of CKD—accounting for approximately 50% of cases of CKD, it is likely that the rise in CKD mortality from 1999–2009 was driven in part by increasing diabetes prevalence during that period. The subsequent decline in CKD mortality from 2009–2012 is more difficult to explain but may reflect delayed benefits from improvements in diabetes treatment in the United States during the early 2000s. Additionally, the lack of continued improvement in CKD mortality among older adults is likely at least in part attributable to stagnation or regression in diabetes care in the United States after 2010. These trends highlight the importance of continued improvement in CKD risk factor control among older adults, and indicate that further interventions are required to reduce the prevalence of diabetes and hypertension among this population.

When examining the primary causes of death among people who died from kidney disease listed as a contributing cause of death, we found that heart disease was the leading cause of death, followed by cancer. This is consistent with existing literature, as cardiovascular disease is well-established as the leading cause of death among people with kidney disease. Several mechanisms underlie this association: cardiovascular and kidney diseases share common risk factors, most importantly diabetes and hypertension [14]. Additionally, renal dysfunction leads to alterations in lipid metabolism that promote atherosclerosis and increase cardiovascular risk [14]. Although cardiovascular disease and cancer are the two leading causes of death in the general population, the relative burden differs notably among people aged 65 and older with kidney disease. In the general population, cardiovascular mortality is approximately 20% higher than mortality from cancer. However, in our study, cardiovascular mortality among individuals with kidney disease was nearly twice that of cancer mortality [48]. These findings emphasize the importance of prevention and management of kidney disease—not only to reduce renal-specific outcomes but also to mitigate the substantial burden of cardiovascular mortality in this high-risk population. Additionally, further research is required to elucidate the mechanisms by which kidney disease contributes to cardiovascular disease, and to improve risk stratification for cardiovascular events among patients with kidney disease.

This study has multiple limitations. First, the study relies on data from CDC WONDER, which are derived from death certificates and may be affected by subjective interpretation when assigning primary and contributing causes of death. Second, due to limitations in earlier versions of the CDC WONDER database, Hispanic ethnicity was not consistently tabulated, we did not analyze trends in deaths from kidney diseases among older Hispanic adults. Third, data on risk factors are limited in the CDC WONDER database and as such we were unable to comprehensively determine how factors such as diabetes and hypertension may have contributed to the risk of kidney disease death and how the risk of kidney disease death may have been directly affected by changes in rates of diabetes and hypertension in the United States population over time. Additionally, data in CDC WONDER is aggregated and as such we did not have access to individual-level data on either risk factors or socioeconomic status. As such we could not construct multivariable analyses controlling for comorbidities and socioeconomic status to better understand the relationship between demographic factors and kidney disease mortality. Finally, this study is specific to the United States—a country with a unique healthcare landscape characterized by high overall wealth, but also significant economic and racial inequality, along with limited healthcare access not commonly seen in other high-income nations [49,50,51]. As such, the generalizability of our findings to international settings may be limited.

Despite these limitations, this study also has multiple strengths. This is the first study to comprehensively examine kidney disease-related deaths among older adults in the United States. Given the aging of the United States population and the fact that the majority of kidney disease-related deaths occur is this age group, this examination is essential for understanding the epidemiology of kidney disease in the United States and for building policy and clinical solutions to this growing public health challenge. CDC WONDER is a nationally representative and comprehensive source of mortality data encompassing all 50 states and the District of Columbia. As such, we were able to build a comprehensive picture of deaths and disparities from kidney disease among the study population. Additionally, to our knowledge this is the first study to analyze trends in kidney disease deaths while including the most recent data up to 2023. Third, our analysis of kidney disease as both a primary and contributing cause of death builds a more complete picture of its role in overall mortality. This dual approach captures not only deaths directly attributed to kidney disease but also those in which it played a contributory role, thereby reflecting its broader impact on public health.

## 5. Conclusions

In summary, our findings highlight a significant mortality burden from kidney disease among older adults in the United States, with particularly elevated rates among Black Americans and those living in the Southern region. These disparities underscore the urgent need for further research into the social, environmental, and structural factors contributing to unequal kidney disease outcomes. As treatment strategies for kidney disease and associated risk factors continue to advance, clinicians and policymakers should prioritize equity-focused approaches. With the ongoing aging of the United States population, improving access to care and treatment for older Americans with kidney disease will be critical to preventing a further rise in this growing mortality burden.

## Figures and Tables

**Table 1:** Mortality from kidney disease as the primary cause of death among older adults by sex, race, urbanization, region, and education 2018–2023.

Variable	Demographic	Number of Deaths	Mortality Rate	Mortality Rate Ratio
Overall		263,436	83.608	
Sex				
	Female	130,492	71.23	Ref
	Male	132,944	101.50	1.42 (1.42–1.43)
Race				
	White	207,548	78.20	Ref
	Blackor African American	44,155	151.41	1.94 (1.93–1.95)
	Asian	8794	58.61	0.75 (0.73–0.77)
	American Indian or Alaska Native	1552	66.77	0.85 (0.80–0.91)
	Native Hawaiian or Other Pacific Islander	402	91.20	1.17 (1.07–1.27)
	More than one race	985	33.27	0.43 (0.36–0.49)
Ethnicity				
	Hispanic	20,648	75.99	Ref
	Non-Hispanic	242,346	84.12	1.11 (1.09–1.12)
Region				
	Northeast	50,789	85.42	Ref
	Midwest	64,519	95.60	1.12 (1.11–1.13)
	South	107,484	90.77	1.06 (1.05–1.07)
	West	40,644	57.95	0.68 (0.67–0.69)

Age-adjusted mortality rates per 100,000.

**Table 2:** Trends (95% CI) in deaths from kidney disease as the primary cause of death among adults aged 65+.

Variable	Demographic	APC Trend 1 ^a^	*p*	APC Trend 2 ^b^	*p*	APC Trend 3 ^c^	*p*	APC Trend 4 ^d^	*p*
Overall		1.40 (0.90, 2.09)	<0.01	−5.35 (−6.59, −2.33)	<0.01	−0.47 (−0.90, 0.40)	0.20		
Sex									
	Female	1.56 (1.09, 2.18)	<0.01	−5.72 (−6.87, −2.80)	<0.01	−0.43 (−0.84, 0.24)	0.18		
	Male	0.96 (0.25, 2.10)	0.03	−5.23 (−6.72, −1.35)	0.03	−0.68 (−1.40, 1.67)	0.29		
Race									
	White	1.54 (0.88, 2.51)	0.02	−5.17 (−6.62, −1.55)	0.03	−0.54 (−1.15, 1.37)	0.32		
	Black	0.73 (0.30, 1.36)	<0.01	−6.65 (−7.80, −3.77)	<0.01	−0.16 (−0.60, 0.50)	0.56		
	Asian	−0.33 (−0.74, 0.09)	0.12						
Region									
	Northeast	2.78 (1.16, 5.15)	<0.01	−0.42 (−1.26, 0.13)	0.11	−5.59 (−6.32, −3.75)	<0.01	−0.29 (−0.53, 0.02)	0.06
	Midwest	4.63 (0.88, 10.26)	0.01	−0.82 (−1.15, −0.60)	<0.01				
	South	2.48 (1.83, 3.31)	<0.01	−5.45 (−6.83, −1.95)	0.03	−1.08 (−1.61, 0.25)	0.07		
	West	1.69 (1.03, 2.70)	<0.01	−6.92 (−8.61, −2.78)	<0.01	1.41 (−0.81, 2.32)	<0.01		

Abbreviations: APC: Annual percent change; CI: confidence interval. ^a^ Table 1. from 1999–2009 for overall population, male, female, White, and Black adults, and in South and West regions. Trend 1 from 1999–2002 in Northeast and Midwest regions. Trend 1 from 1999–2023 for Asian adults. ^b^ Trend 2 from 2009–2012 for overall population, male, female, White, and Black adults, and in South and West regions. Trend 2 from 2002–2009 in Northeast region. Trend 2 from 2009–2023 in West region. ^c^ Trend 3 from 2012–2023 for overall population, male, female, White, and Black adults and in South and West regions. Trend 3 from 2009–2012 in Northeast region. ^d^ Trend 4 from 2012–2023 in Northeast region.

**Table 3:** Mortality from kidney disease as multiple cause of death among older adults by primary cause of death, sex, race, urbanization, region, and education 2018–2023.

Variable	Demographic	Number of Deaths	Mortality Rate	Mortality Rate Ratio
Overall		1,164,575	369.04	
Sex				
	Female	551,981	300.52	Ref
	Male	612,594	465.82	1.55 (1.55–1.55)
Race				
	White	965,178	359.75	Ref
	Black or African American	157,772	537.26	1.49 (1.49–1.50)
	Asian	36,661	243.27	0.68 (0.67–0.69)
	American Indian or Alaska Native	7400	313.88	0.87 (0.85–0.90)
	Native Hawaiian or Other Pacific Islander	1725	392.03	1.09 (1.04–1.14)
	More than one race	4839	162.12	0.45 (0.42–0.48)
Ethnicity				
	Hispanic	89,514	327.42	Ref
	Non-Hispanic	1,072,919	351.56	1.07 (1.07–1.08)
Region				
	Northeast	197,661	332.11	Ref
	Midwest	269,688	399.28	1.22 (1.21–1.23)
	South	452,967	381.81	1.17 (1.16–1.17)
	West	244,259	347.61	1.06 (1.05–1.07)
Primary cause				
	Heart disease	227,542	72.71	Ref
	Cancer	130,756	41.01	0.57 (0.56–0.57)
	COVID-19	84,680	26.53	0.37 (0.36–0.38)
	Chronic Lower Respiratory Diseases	42,016	13.45	0.19 (0.18–0.20)
	Sepsis	29,972	9.40	0.13 (0.12–0.14)
	Cerebrovascular disease	26,871	8.55	0.12 (0.11–0.13)

## Data Availability

All data used in this manuscript is publicly available at wonder.cdc.gov.
